# Associations between the SMARS score derived from CT and MRI with histopathological features in HCC

**DOI:** 10.1038/s41598-025-09918-8

**Published:** 2025-07-10

**Authors:** Daniele Romeo, Theresa Richter, Anne-Kathrin Höhn, Hans-Michael Tautenhahn, Daniel Seehofer, Uwe Scheuermann, Timm Denecke, Hans-Jonas Meyer

**Affiliations:** 1 Department of Diagnostic and Interventional Radiology, University Hospital Leipzig, Liebigstraße 20, 04103, 49341/9717400 Leipzig, Germany; 2 Department of Biomedicine, Neuroscience and Advanced Diagnostics (BiND), University of Palermo, Palermo, Italy; 3 Department of Pathology, University Hospital Leipzig, Leipzig, Germany; 4 Department of Hepatobiliary and Transplantation Surgery, University Hospital Leipzig, Leipzig, Germany

**Keywords:** Cancer, Biomarkers, Oncology

## Abstract

There are complex associations between the imaging phenotype and underlying histopathology of hepatocellular carcinomas (HCC). The recently proposed SMARS score (acronym comprising *S*hape of tumour, *M*osaic architecture, *A*FP level, *R*im APHE, and *S*atellite lesion) could discriminate proliferative and non-proliferative HCC tumours in a non-invasive way and was associated with treatment outcomes. However, a systematic validation of this score is needed and it is unclear whether associations with histopathology features exist. The present study elucidates possible correlations between the SMARS score defined by CT and MRI images with immunohistochemistry features of the pathological specimens in a curatively treated HCC cohort. A total of 44 patients (mean age: 59.6 ± 10.7 years) with histologically confirmed HCC, who underwent curative surgical resection, were included in the present analysis. Contrast enhanced MRI and CT images were performed before surgery and the SMARS score was calculated. The pathological specimens were analyzed for programmed death ligand 1 (PD-L1), Glypican-3, CD3-tumour infiltrating lymphocyte, CD68 positive cells, CD34 positive microvessel density (MVD). The median SMARS score derived from MRI images was 1.4 (interquartile range: -0.32; 2.18) and from CT images it was − 0.32 (interquartile range: -1.08; 0.56). According to the proposed threshold, 29 tumours were categorized as proliferative HCC (82.9%) and six tumours as nonproliferative HCC (17.1%) accordingly to the MRI SMARS score. According to the CT SMARS score 24 tumours were categorized as proliferative HCC (61.5%) and 15 as nonproliferative HCC (38.5%). The SMARS score derived from MRI images showed no correlations with the PD-L1, CD68, CD3 and MVD parameters. However, a moderate association was shown between the SMARS score with the Glypican-3 expression (*r* = 0.37, *p* = 0.03). The SMARS score derived from CT images, instead, showed correlations with two of the PD-L1 parameters (for PD-L1 tumour positive score *r*=-0.37, *p* = 0.02 and for PD-L1 combined positive score *r*=-0.35, *p* = 0.03) while no other association with the remaining parameters was detected. The SMARS score as a promising novel imaging score is associated with the Glypican-3 and PD-L1 expression in curatively treated HCC patients. Differences between the CT and MRI defined score needs to be investigated in further trials on larger patient cohorts.

## Introduction

The diagnosis of hepatocellular carcinoma (HCC) can be made by imaging modalities based on contrast media criteria of arterial hypervascularization and portavenous wash-out phenomena^1–3^. These imaging characteristics represent neoangiogenesis and malignant transformation, allowing to diagnose this cancer type without histopathologic confirmation in half of the cases^1–3^. In the other half of suspicious HCC tumours the biopsy and histological evaluation is still demanded^3^.

The SMARS score, (acronym comprising *S*hape of tumour, *M*osaic architecture, *A*FP level, *R*im APHE, and *S*atellite lesion) comprising the tumour features shape, architecture, AFP level, rim enhancement and satellite lesion was introduced as a potential easy to calculate imaging based score to diagnose proliferative HCC^4^. In a recently published study by Bao et al., investigating overall 1994 patients with HCC, the cutoff value of the SMARS score for the identification of proliferative and nonproliferative HCCs was − 0.49, with a strong predictive accuracy in the training and in the validation cohort^4^. In the original study, the diagnosis of proliferative HCC was made by the subtype of HCC of surgical specimens without further immunohistochemical assessment.

In another study by Zhou et al., the SMARS score was evaluated for treatment outcome after local microwave ablation (MWA)^5^. The study concluded that the predicted proliferative HCCs have worse recurrence free survival than nonproliferative ones after MWA treatment without the need for further histopathological work-up^5^.

It remains unclear, whether this imaging score cannot only predict the treatment outcome of patients undergoing transarterial chemoembolization and microwave ablation but if it is also associated with immunohistochemical features of HCC reflecting the tumour-infiltrating lymphocytes, microvessel density, and immune scores. Notably, it is a well-established fact, that some imaging findings are related to the underlying histopathology of HCC^6–9^.

As the SMARS score is comprised of different phenotypically features defined by imaging, it seems plausible that there is the link between the imaging based phenotypically expression and the underlying histopathology features of the tumours.

Therefore, the purpose of this study was to elucidate possible associations between the imaging based SMARS score derived from CT- and MRI images with immunohistochemical features in surgically resected HCC patients.

## Materials and methods

This retrospective study was approved by the institutional ethic committee (University of Leipzig, approval number: 159/25-ek ) and informed consent was waived.

## Patients and inclusion criteria

Patients were included in this study if they fulfilled the following inclusion criteria: (1) pre-interventional contrast enhanced CT or MRI within 1 year before the surgery, (2) lesion size > 5 mm, (3) available pathologic specimens, and (4) histological proven HCC. Exclusion criteria were: significant artifacts on presurgical CT or MR images.

Altogether, 44 patients, 8 women (18.2%) and 36 men (81.8%) with a mean age of 59.6 ± 10.7 years could be included into the present study.

## MR imaging

In all patients, MRI was performed on a clinical 1.5-T scanner (Aera, Siemens Health Care, Erlangen, Germany). The imaging protocol included T2-weighted single-shot and turbo-spin echo sequences with and without fat suppression (TR/TE: 1600:100). Dynamic contrast-enhanced scans were obtained after administration of Gd-EOB-DTPA (0.1 mmol/kg body weight, Primovist^®^, Bayer HealthCare): T1 weighted gradient echo sequences in the arterial, portal-venous and late venous phase as well as hepatobiliary phase 20 min after contrast media application. The parameters of the sequence were as follows: (TR 4 ms, TE 2 ms, Matrix 172 × 172, Field of view 345 × 345 mm, flip angle 10°.

## CT imaging

CT imaging was performed in a clinical setting with a 256 slice CT scanner (iCT, Philips, Amsterdam, Netherlands) after intravenous application of 90 mL iodinated intravenous contrast medium (injected at a rate of 2 mL/s by a power injector, Medtron GmbH, Germany), with a scan delay of 20 and 70 s after onset of injection for the arterial and portalvenous, respectively. Typical imaging parameters were 120 kVp and 150–300 mAs. Reconstructed slice thickness was 1 mm.

## Imaging analysis

CT and MR images were analyzed within the clinical used PACS. The SMARS score was calculated on CT- and MRI images by a trained resident with 1 year of general experience as well as by a board-certified radiologist with 8 years of general experience. The CT and MRI images were used to independently calculate the score.

**Fig. 1:**
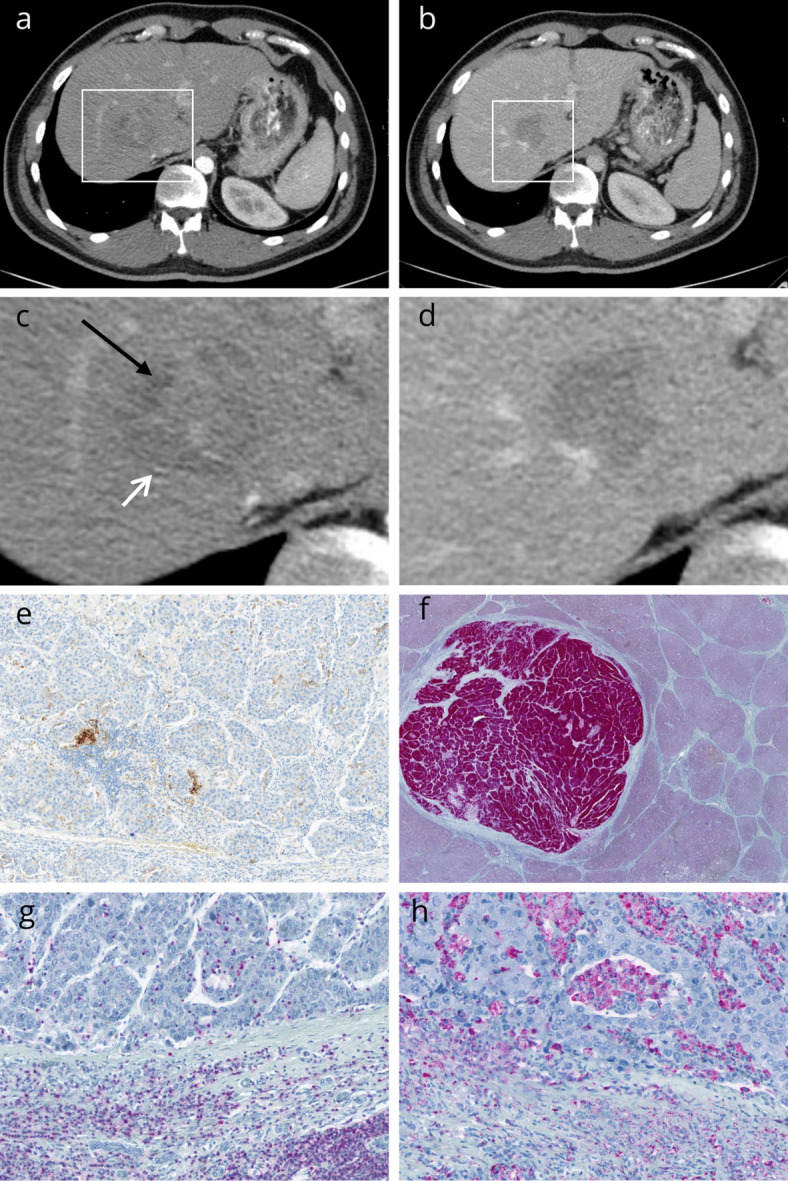
CT images of a patient with a proliferative HCC in the right liver lobe, segment VIII. **A**. Arterial phase with a heterogeneous enhancement and mosaic-pattern. No satellite lesions or lobulated shape or rim APHE. The AFP level was over 200 ng/ml. **B**. Portalvenous phase shows a washout phenomenon of the lesion consistent with the HCC criteria. **C**. and **D**. magnified view of the tumour. In the arterial phase (white arrow and black arrow) feature of mosaic appearance is clearly visible. **E**. PD-L1 staining of the tumour with positive tumour and stroma cells. **F**. Glypican-3 expression of the tumour. **G**. CD3 staining demonstrating tumour and stroma positive infiltrating lymphocytes. **H**. CD68 staining with positivity of both compartments.

In short, the scores included five parameters, *S*hape of tumour, *M*osaic architecture, *A*FP level, *R*im APHE, and *S*atellite lesion^4^. The proposed formula was 0.767 × Shape of tumour + 1.196 × Mosaic architecture + 0.881 × AFP level + 2.506 × Rim APHE + 1.178 × Satellite lesion − 8.811. The proposed cutoff value of − 0.49 based on the prior validation of Bao et al., was used to identify predicted proliferative and nonproliferative HCCs^4^. Figure 1 provides two representative cases of the patient.

## Histological analysis

Histopathology evaluation was performed by one board-certified pathologist (A.K.H.) without knowledge of the patients or imaging data. Formalin-fixed, paraffin-embedded tissue serial Sect. (2 μm) were dewaxed in xylol and rehydrated by descending concentrations of ethanol. For each specimen, standard hematoxylin and eosin (HE) staining and immunohistochemistry were performed. For antigen detection, we used the automated immunohistochemistry slide staining system VENTANA BenchMark ULTRA (Roche Diagnostics GmbH), the VENTANA iVIEW DAB Detection Kit or ultraView Universal Alkaline Phosphatase Red Detection Kit (Roche Diagnostics GmbH) before counterstaining with Haemalaun solution. All tumors were further investigated with the following immunohistochemistry features: programmed death ligand 1 (PD-L1, clone 22C3, dilution 1:100, Dako), Glypican-3 (clone IG12, dilution 1:20, DCS), CD3-tumour infiltrating lymphocyte (polyclonal, dilution 1:150, DAKO), CD68 positive cells (clone PM-M1, dilution 1:100, DAKO) and CD34 positive microvessel density (clone QBEND10, dilution 1:300, Beckmann Coulter).

### Statistical analysis

Statistical analysis and graphics creation were performed using GraphPad Prism 10 (GraphPad Software, La Jolla, CA, USA). Collected data were evaluated by means of descriptive statistics (absolute and relative frequencies). Spearman’s correlation coefficient (r) was used to analyze associations between investigated parameters. For discrimination analysis, the Mann–Whitney-U test was used. The agreement between the MRI- and CT-derived SMARS score was evaluated with intraclass coefficients (ICC). In all instances, p-values < 0.05 were taken to indicate statistical significance.

## Results

The demographics of the patient sample is provided by Table 1. In brief, 38 patients had a liver cirrhosis (86.4%), in half of cases connected to alcohol abuse and in 19 cases (43.1%) due to nonalcoholic fatty liver disease (NAFLD).

**Table 1:** Overview of the characteristics of the investigated study population.

Characteristics (*n* = 44)	Mean value	Percentage
**Mean Age (Years)**	59.6 ± 10.7	
**Male**	36	81.8
**Female**	8	18.2
**Cirrhosis**		
**Present**	38	86.4
**Absent**	6	13.6
**Tumour size (cm)**	3.2 ± 1.9	
**Underlying Liver Disease**		
**Alcoholic cirrhosis**	10	22.7
**Alcoholic cirrhosis and DM2**	9	20.5
**DM2**	8	18.2
**No pathologies reported**	7	15.9
**Alcohol**,** DM2**,** HCV**	2	4.6
**HBV**	2	4.6
**HCV**	1	2.3
**HBV**,** HCV**,** HDV coinfection**	1	2.3
**HBV**,** HCV**,** HDV coinfection**,** alcohol**	1	2.3
**Other causes**	3	6.8
**Histopathologic Features**	**Mean Value**	**Standard deviation**
**PD-L1 ICS**	0.59	1.3
**PD-L1 TPS**	0.41	1.6
**PD-L1 CPS**	0.45	1.1
**Glypican3**	1.64	0.8
**CD3 TIL**	2.05	0.9
**CD3 SIL**	3.37	0.9
**CD68 intratumoural**	1.23	0.4
**CD68 peritumoural**	1.39	0.5
**CD34 MVD**	42.5	11.8

cm centimetre, DM2 diabetes mellitus, HBV hepatitis B virus, HCV hepatitis C virus, HDV hepatitis D virus, PD-L 1 programmed death ligand 1, ICS immune cell score, TPS tumour positive score, CPS combined positive score, TIL tumour infiltrating lymphocyte, SIL stroma infiltrating lymphocyte, MVD microvessel density.

For five patients (11.4%) only MRI images were available and for nine patients (20.5%) only CT images and for the remaining 30 patients (68.1%) both imaging modalities were available for evaluation and SMARS score was calculated for both CT and MRI imaging. Mean time period between the imaging and histopathology evaluation is 5.2 months for the MRI and 6.2 for the CT imaging, respectively. The mean time period for patients with both modalities is 2.3 months with a shorter time frame for the MRI.

**Fig. 2:**
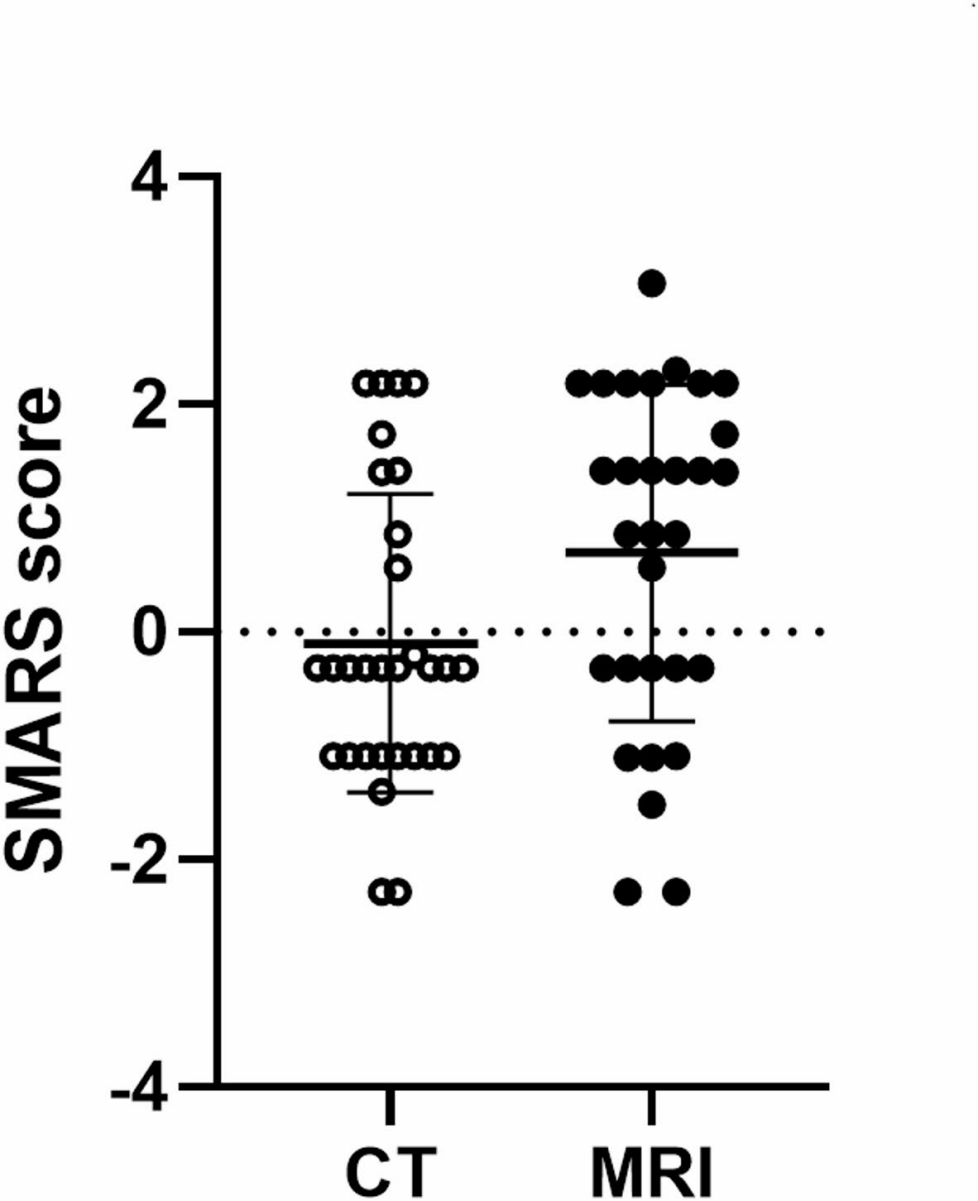
Scatter blot showing the SMARS Score of CT and MRI images. The MRI-derived score was slightly higher compared to the CT-derived score (*p* = 0.046, Mann-Whitney test).

The median SMARS score derived from MRI images was 1.4 (interquartile range: −0.32; 2.18) and from CT images it was − 0.32 (interquartile range: −1.08; 0.56), *p* = 0.046 (Fig. 2).

Table 2 provides the imaging characteristics of the SMARS score divided by CT and MRI images. According to the proposed threshold 29 tumours of the 35 included cases in the MRI analysis were categorized to proliferative HCC (82.9%) and 6 tumours nonproliferative (17.1%) accordingly to the MRI SMARS score. For the CT SMARS score, 24 of the 39 included cases (61.5%), were proliferative HCC and 15 (38.5%) were nonproliferative HCC. Table 3 provides the SMARS score results divided by CT and MRI images.

**Table 2:** Imaging characteristics of the SMARS score stratified by MRI and CT.

SMARS Score features - MRI	Absent (%)	Present (%)
**Lobulated Shape**	13 (37.1)	22 (62.9)
**Satellite Lesions**	27 (77.1)	8 (22.9)
**Rim APHE**	18 (51.4)	17 (48.6)
**Mosaic Architecture**	6 (17.1)	29 (82.9)
**AFP (200 ng/ml)**	31 (88.6)	4 (11.4)
**AFP (200 ng/ml)**	35 (89.7)	4 (10.3)

**SMARS score features - CT**		
**Lobulated Shape**	18 (46.1)	21 (53.9)
**Satellite Lesions**	36 (92.3)	3 (7.7)
**Rim APHE**	32 (82.1)	7 (19.9)
**Mosaic Architecture**	6 (15.4)	33 (84.6)
**AFP (200 ng/ml)**	35 (89.7)	4 (10.3)

**Table 3:** Performance of the SMARS score per CT and MRI imaging side-by-side.

	Number of Patients	%
**MRI**	35	
**CT**	39	
**Proliferative in MRI Imaging**		
**Yes**	29	82.9
**No**	6	17.1
**Proliferative in CT Imaging**		
**Yes**	24	61.5
**No**	15	38.5
**Mean SMARS Score**	Median	IQR
**MRI**	1.4	−0.32;2.18
**CT**	−0.32	−1.08;0.56

There was only a moderate correlation between the CT-derived and the MRI-derived SMARS score (*r* = 0.45, *p* = 0.0019). The intraclass coefficient between the scores demonstrated a moderate agreement with 0.57 (95% CI 0.10–0.79, *p* = 0.01).

## Correlation analysis

The results of the correlation analysis are summarized in Tables 4 and 5. There was no correlation between the tumour size and the MRI-derived SMARS score (*r* = 0.21, *p* = 0.23) nor with the CT-derived SMARS score (*r* = 0.29, *p* = 0.07).

**Table 4:** Spearman’s correlation analysis between the MRI derived SMARS score and the histopathology features.

PD-L1 ICS	PD-L1 TPS	PD-L1 CPS	Glypican-3	CD3 TIL	CD3 SIL	CD68 tumoural	CD68 peritumoural	MVD
*r*= −0.27, p= 0.11	*r* = 0.06, p= 0.74	*r*= −0.08, *p* = 0.64	**r=** **0.37**,** p = 0.03**	*r*= −0.17, *p* = 0.33	*r*= −0.28, *p* = 0.11	*r* = 0.025, *p* = 0.89	*r* = 0.07, *p* = 0.67	*r*= −0.26, *p* = 0.14

**Table 5:** Spearman’s correlation analysis between the CT derived SMARS score and the histopathology features.

PD-L1 ICS	PD-L1 TPS	PD-L1 CPS	Glypican-3	CD3 TIL	CD3 SIL	CD68 tumoural	CD68 peritumoural	MVD
*r*=−0.30, p= 0.06	**r=** **−0.37**,** p= 0.02**	**r=** **−0.35**,** p=** **0.03**	*r* = 0.05, *p* = 0.77	*r*= −0.04, *p* = 0.80	*r*= −0.035 , *p* = 0.83	*r* = 0.075, *p* = 0.65	*r*= −0.09, *p* = 0.58	*r* = 0.11 *p* = 0.51

**Fig. 3:**
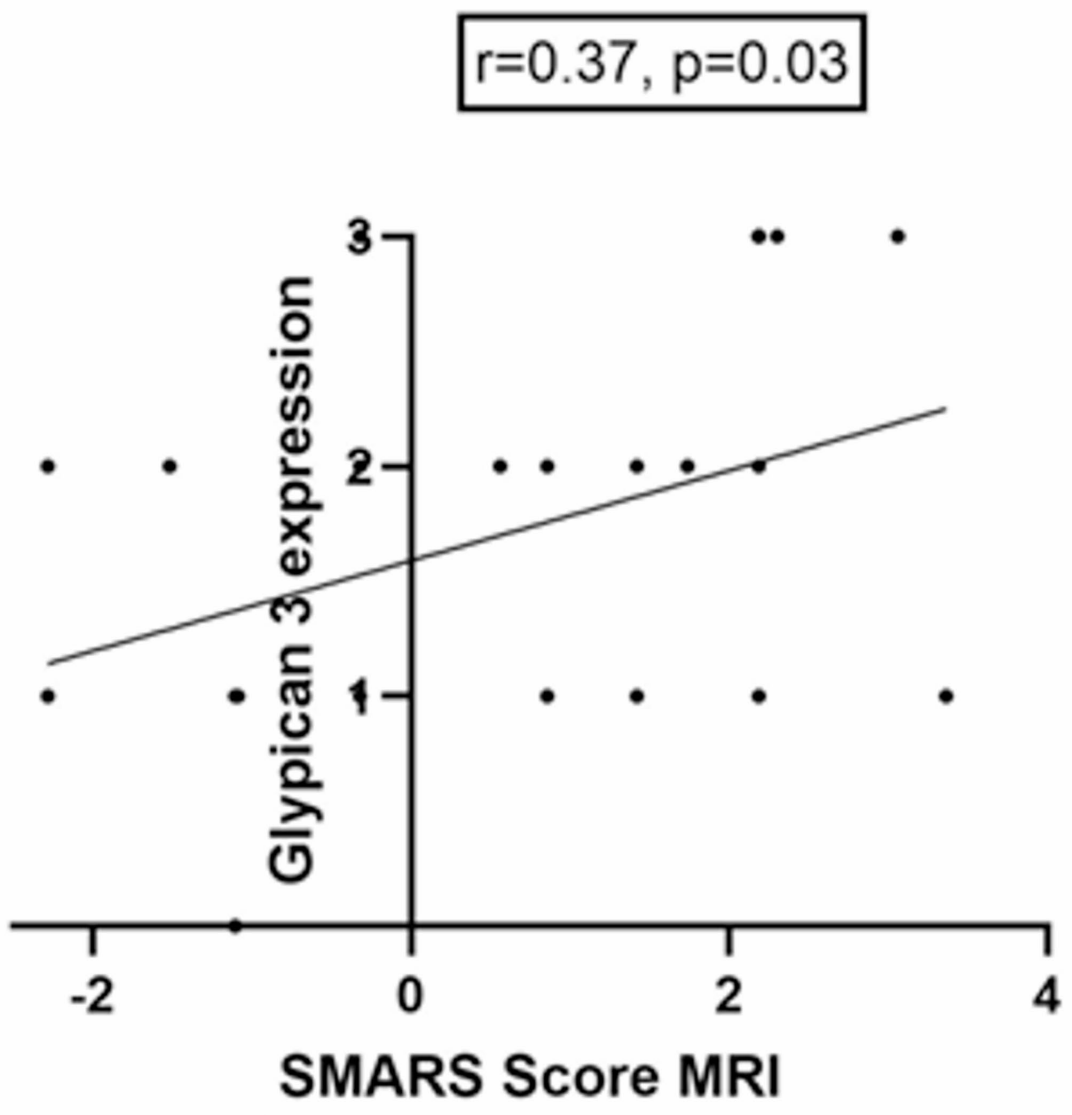
Correlation graph of the MRI-derived SMARS Score and Glypican-3 expression with a correlation coefficient of *r* = 0.37, *p* = 0.03. Spearman’s correlation was used, p-value < 0.05 was taken to indicate statistical significance.

The SMARS score derived from MRI images showed no correlations with the PD-L1, CD68, CD3 and MVD parameters. However, a moderate association was shown between the SMARS score with the Glypican-3 expression (*r* = 0.37, *p* = 0.03). The corresponding graph is shown in Fig. 3.

**Fig. 4:**
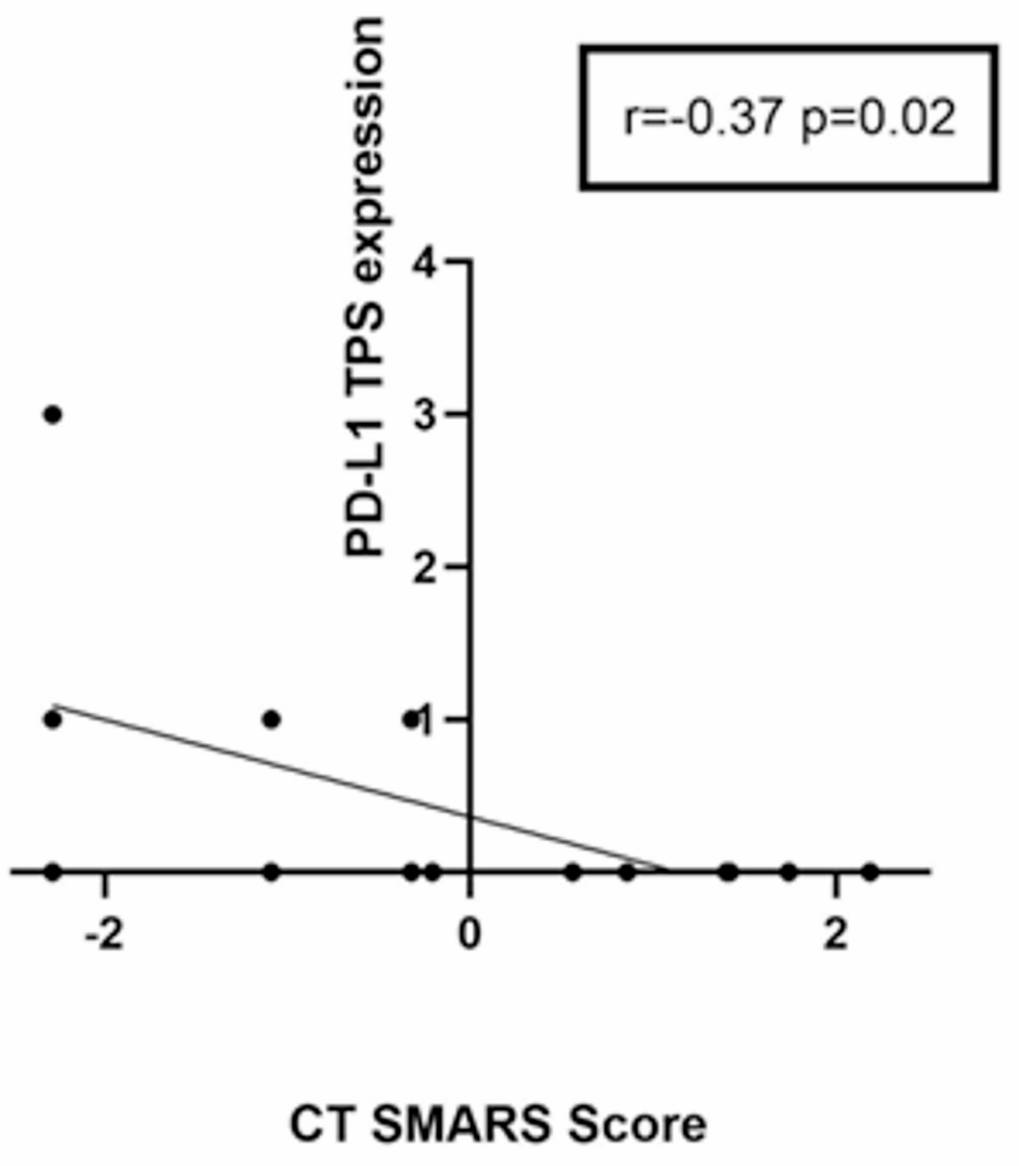
Correlation graph of the CT-derived SMARS Score and PD-L1 tumour positive score expression with a correlation coefficient of *r*=−0.37, *p* = 0.02. Spearman’s correlation was used, p-value < 0.05 was taken to indicate statistical significance.

**Fig. 5:**
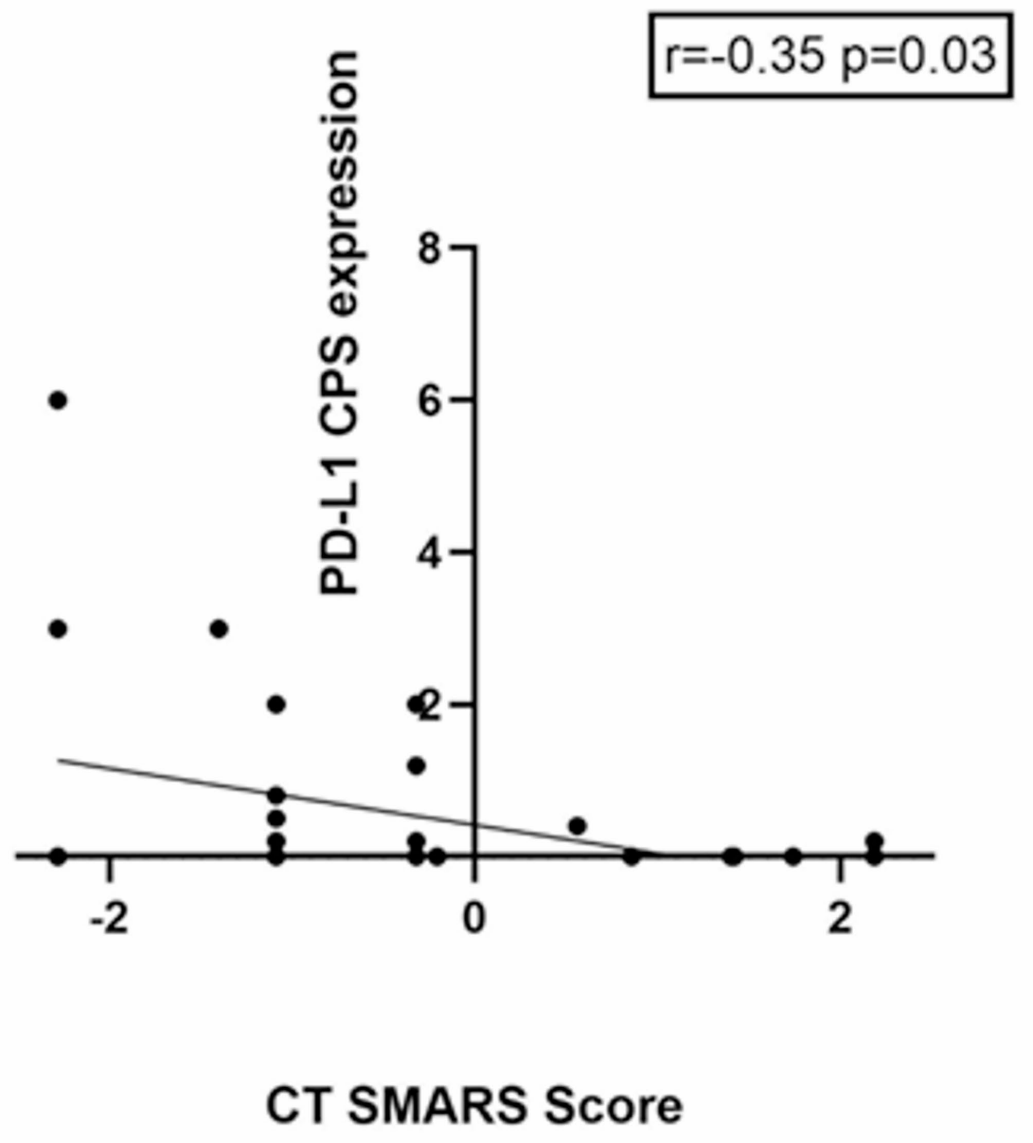
Correlation graph of the CT-derived SMARS Score and PD-L1 combined positive score expression with a correlation coefficient of *r*=−0.35, *p* = 0.03. Spearman’s correlation was used, p-value < 0.05 was taken to indicate statistical significance.

The SMARS score derived from CT images showed correlations with the investigated immunohistochemical features: PD-L1 TPS (*r*=−0.37, *p* = 0.02) and PD-L1 CPS (*r*=−0.35, *p* = 0.03) (Figs. 4, 5).

## Discrimination analysis

The results of the discrimination analysis for proliferative versus nonproliferative HCC are presented by Table 6and,7. The PD-L1 CPS showed a significant difference between the two groups in the CT SMARS score analysis with a p-value of 0.04.

**Table 6:** Discrimination analysis between proliferative and nonproliferative HCC defined by MRI.

Parameter	Proliferative HCC (*n* = 29)	Nonproliferative HCC (*n* = 6)	*p*-value
**PD-L1 ICS**	0.45 ± 1.0	1.17 ± 1.9	0.25
**PD-L1 TPS**	0.48 ± 1.8	0.50 ± 1.2	0.99
**PD-L1 CPS**	0.36 ± 0.7	1.12 ± 2.4	0.44
**Glypican-3**	1.86 ± 0.8	1.17 ± 0.7	0.11
**CD3 TIL**	1.97 ± 0.8	2.5 ± 0.5	0.21
**CD3 SIL**	3.43 ± 0.8	3.5 ± 0.5	0.99
**CD 68 tumoural**	1.17 ± 0.3	1.3 ± 0.5	0.58
**CD 68 peritumoural**	1.35 ± 0.4	1.5 ± 0.5	0.65
**MVD**	43.45 ± 11.7	50.0 ± 10.9	0.19

**Table 7:** Discrimination analysis between proliferative and nonproliferative HCC defined by CT.

Parameter	Proliferative HCC (*n* = 24)	Nonproliferative HCC (*n* = 15)	*p*-value
**PD-L1 ICS**	0.25 ± 0.5	1.33 ± 1.9	0.05
**PD-L1 TPS**	0.08 ± 0.2	1.0 ± 2.6	0.11
**PD-L1 CPS**	0.17 ± 0.4	1.03 ± 1.7	**0.04**
**Glypican-3**	1.75 ± 0.8	1.47 ± 0.9	0.37
**CD3 TIL**	2.0 ± 0.8	2.13 ± 0.7	0.72
**CD3 SIL**	3.35 ± 1.0	3.53 ± 0.5	0.99
**CD 68 tumoural**	1.29 ± 0.4	1.20 ± 0.4	0.71
**CD 68 peritumoural**	1.42 ± 0.5	1.47 ± 0.5	0.99
**MVD**	41.25 ± 11.1	41.33 ± 13.0	0.93

No immunohistochemical parameter showed a difference between the group of proliferative and nonproliferative HCC according to the SMARS score threshold in the MRI SMARS score analysis.

Regarding the imaging features of the SMARS score, lobulated shape tumours defined by MRI had statistically higher PD-L1 TPS values (0.77 ± 2.2) compared to non-lobulated tumours (0, *p* = 0.03). The amount of CD3 TIL were lower in the non-lobulated tumours (2.5 ± 0.5 versus 1.8 ± 0.8, *p* = 0.008). For the CT-defined features, tumours with mosaic pattern had a higher PD-L1 TPS score compared to the non-mosaic tumours (2.3 ± 3.9 versus 0.09 ± 0.3, *p* = 0.009).

## Discussion

The present study evaluated possible associations between imaging-based SMARS score with immunohistochemical features in curatively treated HCC. The SMARS score derived from MRI images showed a moderate correlation with Glypican-3 expression, indicating that it the MRI phenotype is linked to this important cell membrane associated heparan sulfate proteoglycan. However, there were no association between the SMARS score with PD-L1, tumour-infiltrating lymphocytes and microvessel density. The CT-defined SMARS score demonstrated an inverse correlation with the PD-1 scores TPS and CPS, indicating a possible role in the immune modulation of the tumour.

Previous studies showed that imaging modalities, especially contrast enhanced imaging can reflect tumour biology in HCC^1–3^. The imaging hall marks of HCC are the hypervascularization in arterial phase and the portalvenous wash out phenomena, which are directly linked to the tumour aggressiveness and tumour neoangiogenesis^1–3^. Therefore, it appears plausible that imaging characteristics of dynamic-contrast enhanced CT and MRI should also be able to reflect the investigated histopathology parameters in HCC^1,2,9,12–14^^3,8^. Moreover, the SMARS score reflect distinctive imaging features, which were previously shown to be predictors of HCC likeliness and aggressiveness^4,5^.

Beyond that, the SMARS score includes the shape of the tumour, mosaic architecture, rim arterial phase hyperenhancement (APHE), and satellite lesions, which can be easily used in clinical routine, as they are also included in the Li-RADS lexicon^10,11^. In the first study describing this score by Bao et al., CT images were used to divide the HCC according to their SMARS score into proliferative and nonproliferative groups. However, no direct correlation analysis between the SMARS score and actual immunohistochemical features of the HCC was performed in this study^4^. Moreover, there may be a distinctive difference between the present cohort and the investigated HCC cohort reported by Bao et al. In our study most HCCs were classified as proliferative HCC (61.5%) in CT scan, whereas in the study from Bao et al. only 32% and 31% in the were classified as proliferative HCC in the training and validation cohorts, respectively^4^. It should be discussed that these distinctive differences should not only be caused of the already known differences between western and Asian population HCC with different underlying liver disease but also due to different tumour biologies. Notably, the SMARS score was then transferred to HCC patients undergoing transarterial chemoembolization. This is in contrast to the current patient cohort, which was only treated by surgical resection.

The present analysis showed that the SMARS score differs, when defined by MRI or CT images. In the two abovementioned studies, the score was only evaluated on CT images due to its high availability and standardisation. Yet, it seems logical that the score can also be calculated on MRI images. However, it should be taken into account that MRI images have a better resolution which could result in more patients with mosaic pattern and satellite lesions. We could show that some features of the score are different in MRI images due to its higher resolution and better imaging quality. Beyond that, we could show that the score has different abilities to predict immunohistochemical features of the HCC tumours, when derived from CT or MRI images. Presumably, the SMARS score, originally validated for a CT scan applications, does not include important MRI features, in particular the DWI sequences, which could harbor important aspects of the tumour histopathology and its microenvironment. This finding needs further evaluation in future research.

In recent years, a lot of studies have investigated the association between MRI findings and histopathological features for aggressiveness like microvascular invasion^6–8,15^. For example, a non-smooth tumour margin and incomplete capsule were identified to be predictive for microvascular invasion^16^. The present study further elucidates the associations between the histopathology features and imaging phenotype defined by the SMARS score.

The histopathology parameters investigated in the present study include immune tolerance and tumour-immune microenvironment comprised by tumour-infiltrating lymphocytes, macrophages and PD-L1 expression. Moreover, microvessel density was evaluated as an important factor for the invasiveness of the tumours.

We also evaluated Glypican-3 expression, a heparan sulfate proteoglycan expressed on the surface of HCC cells but not on physiological liver cells. Glypican-3 can not only be used as a biomarker for diagnosis, but also as an important target for immunotherapy of HCC^17^. Notably, Glypican-3 is associated with several different pathways of tumourigenesis including recruitment of immune cells, glucose metabolism and epithelial–mesenchymal transition^17,18^. However, the non-invasive prediction of Glypican-3 expression by imaging modalities is still under investigation. A large study from China could demonstrate that five imaging features, including tumour size > 3.0 cm (OR = 0.5, −3 points), nonperipheral “washout” (OR = 3.0, five points), infiltrative appearance (OR = 9.3, 10 points), marked diffusion restriction (OR = 3.3, five points), and iron sparing in solid mass (OR = 0.2, −7 points) were significantly associated with positive Gypican-3 expression^19^. The authors could show a good prediction model with an AUC 0.726^19^. Another study demonstrated that primarily the apparent diffusion coefficient derived from diffusion-weighted imaging is associated with the Glypican-3 expression^20^. The present study is the first on a Western HCC population to link the associations between MRI findings and Glypican-3 expression in a systematic manner.

There is no doubt regarding the predictive and prognostic importance of PD-L1 expression in HCC especially to guide immunotherapy in a palliative treatment setting but also in curative cases undergoing resection^21–23^. First promising studies have already shown associations between quantitative imaging findings and PD-L1 expression in HCC^24,25^. The present study adds to the identified associations between the SMARS score, TPS and CPS score for a more clinically feasible approach to reflect the PD-L1 expression in clinical routine.

PD-L1 and Glypican-3 are key elements of the tumour microenvironment (TME) definition that depicts a complex interaction between tumour cells and other cell types, increasingly addressed as therapeutic target in HCC^26–28^. PD-L1 expressed in tumour cells, in particular, is involved in the immune surveillance evasion, binding the cell-surface receptor PD-1 expressed by T cells and generating an inhibitory signal that attenuates the activity of T cells^29^. It is, however, recognised the wide distribution, form of expression and ways in which PD-L1 acts in the immune modulation constituting a wide range of elements that needs to be elucidated in order to achieve advancements in cancer immunotherapy^30^.

One study has identified a correlation between imaging and PD-L1 involving radiomics features in PET/CT in patients with NSCLC^31^ . Notably, in a study from Liu et al., it was highlighted how radiomics MRI features, as well as conventional imaging findings such as larger size, non-smooth margins, nodule in nodule, mosaic architecture and corona hyperenhancement were independent factors for microvascular invasion in patients with HCC out of the Milan criteria^32^. Only few of them are part of the SMARS score and this could constitutes a potential explanation on why in our study no significant correlations with MVD were found. However, radiomics analyses requires more advanced technologies and time efforts compared to the SMARS score, which could reduce the possibility of translation into the clinical setting.

The merit of this present study to investigate whether the SMARS score can also be evaluated by MRI images and not only CT images. Notably, the MRI-based score was superior compared to the CT based score. Another merit is that this study tried to link the SMARS score to immunohistochemical features and the underlying tumour biology of the HCC compared to the original description of the study. Further studies are needed to elaborate the interesting implications of the SMARS score in clinical routine.

The associations between the SMARS score and immunohistochemical features such as PD-L1 and Glypican-3, if validated further, could have a clinical utility in a multidisciplinary context. Understanding the correlation with HCC imaging could enhance therapeutic aspects, as integration of imaging-derived immune profiling becomes increasingly relevant for surgical oncologists.

However, one should also acknowledge the only weak to moderate associations between the imaging based SMARS score with the investigated immunohistochemical features. The present results could lead to only a modest accuracy to predict clinically relevant threshold values of the investigated histopathology features. There is need for a prospective external validation in this regard to evaluate the clinical translation of the SMARS score.

The present study is not free from limitations and these promising results must be interpreted considering them. First, it is a retrospective, single-center study with a small sample size. Further validation cohorts based on a prospective multicentric design are needed to explore the complex interactions between imaging and histopathology in HCC. The potential small statistical power of the current study has to be acknowledged.

To overcome some bias, imaging and histopathology assessment was performed blinded to each other. Second, only curatively treated HCC lesions were investigated in the current analysis as histopathological evaluation was only available for these patients, this underscore the need of expanding the study on palliative treated cohorts, as they may significantly differ their tumour biology. Yet, the current study design was directed on the correlation between imaging and histopathology. In palliative HCC patients only few histological specimens are obtained, which limits the possibility of the inclusion of the current study. Therefore, it still remains unclear whether the SMARS score can reflect the immunohistochemical parameters in palliative treated patient cohorts. Third, the 12 months maximal interval between imaging and the pathologic specimens could lead to a potential bias as the tumour has biologically already progressed compared to the imaging time point. However, this also reflects clinical routine with potential time delays between the preoperative imaging and the following resection. Fourth, there is a small time interval between the two imaging modalities in the patients undergoing both imaging modalities. This could also lead to the identified differences of the CT- and MRI-derived SMARS scores but the correlation analyses for both scores were performed independently to the underlying histopathology.

## Conclusion

The SMARS score as a promising novel imaging score is associated with the Glypican-3 expression and PD-L1 expression in curatively treated HCC patients. There are some small differences between the CT and MRI defined score, which needs to be investigated in further trials on larger patient cohorts.

## Data Availability

The anonymized patient data can be provided by the corresponding author upon reasonable request.

## Abbreviations

APHE: arterial phase hyperenhancement
AUC: area under the curve
CT: computed tomography
HCC: hepatocellular carcinoma
ICC: intraclass coefficient
MRI: magnetic resonance imaging
ROC: receiver operating characteristics
ROI: region of interest
